# Autoimmune and Inflammatory Diseases Associated With Increased Periprosthetic Joint Infection Risk: A Narrative Review of Pathophysiology, Risk Stratification, and Perioperative Management

**DOI:** 10.7759/cureus.111079

**Published:** 2026-06-18

**Authors:** Joseph Salem-Hernández, Shakira Bou-Rolón, Peter A Santiago-Gadea, Camilla A Pérez-Vicente, Norman Ramírez

**Affiliations:** 1 Department of Orthopaedic Surgery, Ponce Health Sciences University, Ponce, PRI; 2 Department of Dermatology, Ponce Health Sciences University, Ponce, PRI; 3 School of Medicine, Central University of the Caribbean, Bayamon, PRI

**Keywords:** autoimmune skin diseases, biologic therapy, immunosuppression, perioperative management, periprosthetic joint infection, psoriasis, rheumatoid arthritis, systemic lupus erythematosus

## Abstract

Periprosthetic joint infection (PJI) is a devastating complication of total joint arthroplasty. Patients with autoimmune and inflammatory diseases, psoriasis, rheumatoid arthritis (RA), and systemic lupus erythematosus (SLE) face substantially elevated infection risk through three convergent mechanisms: skin barrier dysfunction, systemic immune dysregulation, and the immunosuppressive therapies required to control their disease. This narrative review synthesizes evidence from cohort studies, registry analyses, mechanistic investigations, and clinical practice guidelines to examine PJI pathophysiology, comparative risk quantification, microbiologic profiles, and perioperative management strategies in this high-risk population. RA patients demonstrated hazard ratios of 4.08 (95% CI 1.35-12.33) and odds ratios of 1.47 (95% CI 1.13-1.91) for PJI compared with osteoarthritis controls; psoriasis patients showed an odds ratio of 1.63 (p = 0.014) for 90-day PJI after total shoulder arthroplasty; and SLE patients carried a hazard ratio of 2.74 (95% CI 1.14-6.64) for late PJI after total hip arthroplasty. These estimates derived from heterogeneous studies and were presented descriptively rather than as pooled values. Perioperative continuation of biologic therapy increased PJI odds by 3.46-fold (95% CI 1.11-10.78), and chronic glucocorticoid use exceeding 10 mg/day approximately doubled infection risk. The microbiologic profile was distinct, with culture-negative infection reported in roughly 28%-38% of cases in a single-center case series of RA patients and methicillin-resistant *Staphylococcus aureus* (MRSA) colonization conferring an odds ratio of 3.43 (95% CI 1.71-6.88) for metachronous PJI. Evidence-based perioperative management required temporarily interrupting biologic agents with surgery scheduled at the end of the dosing cycle, continuing methotrexate in most patients, and applying individualized medication adjustments through multidisciplinary collaboration between orthopedic surgery, rheumatology, and infectious disease.

## Introduction and background

Periprosthetic joint infection (PJI) remains one of the most challenging complications in modern orthopedic surgery, affecting 1% to 2% of primary total joint arthroplasties and up to 4% of revision procedures in the general population [1]. The clinical and economic burden is immense, with individual cases costing between $50,000 and $100,000 and aggregate annual costs in the United States exceeding $1 billion. Treatment typically requires multiple surgical interventions, prolonged antibiotic therapy lasting weeks to months, extended rehabilitation, and, in severe cases, amputation or permanent resection arthroplasty. Even with optimal treatment, success rates for infection eradication range from 70% to 90% depending on organism, timing, and host factors.

For patients with autoimmune and inflammatory diseases, the risk of PJI escalates. This review focuses on psoriasis, rheumatoid arthritis (RA), and systemic lupus erythematosus (SLE). We selected these three conditions because they are the autoimmune or inflammatory diseases for which arthroplasty- and PJI-specific outcome data are most robust, drawing on large cohort, registry, and matched studies. It is important to note that RA and SLE are systemic autoimmune diseases rather than primary dermatologic disorders, and psoriasis is the principal skin disease discussed; we therefore frame the review around autoimmune and inflammatory disease broadly rather than “autoimmune skin disease” as a category. Primary autoimmune dermatologic disorders such as pemphigus vulgaris, bullous pemphigoid, and dermatomyositis are not addressed here because direct arthroplasty and PJI outcome data for them are sparse; these represent an important area for future investigation.

Psoriasis, RA, and SLE affect millions of patients worldwide and frequently necessitate total joint arthroplasty due to progressive joint destruction or avascular necrosis. Although the mechanisms differ by disease, these conditions can converge to increase infection susceptibility through systemic immune dysregulation affecting both innate and adaptive immunity, the requirement for chronic immunosuppressive therapy, and, in psoriasis specifically, disruption of the skin barrier. The pathogenesis of PJI involves bacterial adherence to the prosthetic surface, biofilm formation, and evasion of host immune defenses. Biofilms represent structured bacterial communities encased in a self-produced extracellular polymeric matrix that resists both antibiotic penetration and immune cell killing [1]. This biofilm-mediated immune evasion is particularly problematic in immunocompromised hosts, where baseline defects in neutrophil and macrophage function are further exploited by the biofilm microenvironment.

Recent large-scale cohort studies and registry analyses have quantified the magnitude of PJI risk in these populations, revealing hazard ratios exceeding 4.0 in some RA cohorts and significant elevations in psoriasis and SLE patients [2,3]. The 2022 American College of Rheumatology/American Association of Hip and Knee Surgeons (ACR/AHKS) guideline represents the most recent evidence-based framework for perioperative antirheumatic medication management, though many recommendations remain conditional due to limited high-quality evidence [4].

Methods

This is a narrative review rather than a systematic review or meta-analysis. A narrative literature search was conducted using PubMed and Excerpta Medica database (Embase), supplemented by clinical practice guidelines, for English-language publications addressing PJI in patients with autoimmune and inflammatory disease, with emphasis on contemporary evidence (approximately 2008-2025). Search terms combined concepts of PJI or prosthetic joint infection with RA, psoriasis, psoriatic arthritis, SLE, biologic therapy, immunosuppression, and perioperative management. Cohort studies, registry and claims-database analyses, case-control studies, systematic reviews and meta-analyses, clinical guidelines, and selected illustrative case reports were included; studies were selected for relevance to pathophysiology, risk quantification, microbiology, or perioperative management. Because this is a narrative review, a formal PRISMA flow diagram, structured risk-of-bias scoring of individual studies, and quantitative pooling were not performed; effect estimates are summarized descriptively. This limits formal reproducibility, and conclusions should be interpreted accordingly.

Epidemiology

Traditional risk factors for PJI in the general population include obesity, diabetes mellitus, smoking, malnutrition, prior surgery at the same site, prolonged operative time, and wound complications. Autoimmune and inflammatory diseases and immunosuppressive therapy represent particularly potent risk modifiers that amplify baseline infection risk through multiple mechanisms. The incidence of PJI has remained relatively stable despite advances in surgical technique and infection control, likely reflecting the increasing complexity of patients undergoing arthroplasty and the growing prevalence of immunocompromising conditions. A substantial proportion of arthroplasties in inflammatory-arthritis populations is performed for inflammatory joint destruction or corticosteroid-related avascular necrosis rather than primary degenerative disease, although precise population-level denominators for the annual volume of arthroplasty attributable to inflammatory versus degenerative indications are not well established in the literature.

Autoimmune and inflammatory diseases: clinical overview

RA is a chronic systemic autoimmune disease affecting approximately 0.5% to 1% of the population worldwide, characterized by symmetric polyarticular synovitis leading to progressive joint destruction. The systemic inflammatory nature of RA, combined with the frequent requirement for immunosuppressive therapy, creates a high-risk scenario for postoperative infections, including PJI.

Psoriasis is a chronic inflammatory skin disease affecting 2% to 3% of the population, characterized by erythematous plaques with silvery scales resulting from keratinocyte hyperproliferation and immune dysregulation. Approximately 30% of psoriasis patients develop psoriatic arthritis, an inflammatory arthropathy that can lead to progressive joint damage requiring arthroplasty. Even in patients undergoing arthroplasty for other indications, psoriasis confers elevated PJI risk through skin-barrier dysfunction, altered cutaneous microbiome, and systemic immune abnormalities [3]. Disease severity and phenotype are clinically relevant: body-surface-area involvement, nail psoriasis, inverse psoriasis, scalp psoriasis, and active plaques located at or near the operative site may each be pertinent to surgical infection risk. To our knowledge, the cited psoriasis arthroplasty literature has not stratified PJI risk by psoriasis severity, phenotype, or lesion location, and severity-stratified PJI data in psoriasis are currently lacking.

SLE is a multisystem autoimmune disease with a strong female predominance, characterized by the production of autoantibodies against nuclear antigens, immune complex deposition, and complement activation. SLE patients may require arthroplasty due to inflammatory arthritis, avascular necrosis related to corticosteroid therapy, or secondary osteoarthritis. The profound immune dysregulation in SLE, including complement deficiencies and impaired neutrophil function, combined with chronic immunosuppressive therapy, creates substantial infection risk [5]. All three conditions share chronic systemic inflammation and the requirement for immunosuppressive medications, including corticosteroids, conventional disease-modifying antirheumatic drugs (DMARDs), and biologic agents, though the contribution of skin-barrier disruption and cutaneous microbiome alteration is specific to psoriasis.

## Review

Pathophysiology

The elevated risk of PJI in patients with autoimmune and inflammatory diseases results from the convergence of three mechanisms whose relative importance differs by disease: dermatologic skin-barrier dysfunction (principally relevant to psoriasis), systemic immune dysregulation, and the effects of immunosuppressive therapy. These mechanisms are addressed in turn below, with attention to their disease-specific applicability.

Skin-barrier dysfunction

Skin-barrier dysfunction is directly relevant to psoriasis but applies inconsistently across the other conditions. In psoriasis, epidermal hyperproliferation and abnormal keratinocyte differentiation result in a disrupted stratum corneum with impaired barrier function. Psoriatic plaques demonstrate increased transepidermal water loss and provide potential portals of entry for pathogenic bacteria. The inflammatory milieu, characterized by elevated tumor necrosis factor-alpha (TNF-α), interleukin-17 (IL-17), and interleukin-23 (IL-23), further disrupts barrier homeostasis. Several studies suggest altered microbial composition and increased *Staphylococcus aureus* colonization in subsets of psoriasis patients, which may facilitate pathogen colonization and potential translocation to surgical sites. By contrast, RA patients do not have a primary skin disease; skin fragility, when present, is secondary to chronic inflammation and corticosteroid therapy rather than an intrinsic barrier defect. In SLE, cutaneous involvement is variable, and vascular manifestations such as Raynaud's phenomenon may impair tissue perfusion and healing at surgical sites in some patients. Disease activity in the skin is itself relevant: active psoriatic lesions near the incision may independently increase infection risk, and elective surgery is generally best timed to periods of skin disease control when feasible.

Systemic immune dysregulation

Patients with autoimmune and inflammatory diseases demonstrate systemic immune dysregulation affecting both innate and adaptive immunity. Neutrophils and macrophages serve as the first line of defense against bacterial pathogens, and their function is compromised in multiple conditions. In RA, neutrophils demonstrate impaired chemotaxis, phagocytosis, and bacterial killing capacity, while macrophages show altered polarization and impaired ability to clear bacterial pathogens; these defects are intrinsic to the disease process and are further exacerbated by immunosuppressive medications [1]. Biofilm-associated bacteria evade immune recognition through physical shielding by the extracellular matrix, altered metabolic states, and active suppression of immune cell function, making host defense particularly difficult in patients with baseline immune defects [1]. SLE patients demonstrate particularly severe immune dysregulation, including deficiencies in complement components C1q, C2, and C4 that impair opsonization and bacterial clearance; these factors contribute to the elevated hazard ratio of 2.74 for late PJI observed in SLE patients undergoing total hip arthroplasty [5]. Adaptive immune dysfunction, including T-cell and B-cell abnormalities, further impairs effective responses to bacterial pathogens. The systemic nature of immune dysregulation means infection risk extends beyond the immediate surgical site, with hematogenous seeding of prosthetic joints from distant sources representing a particular concern in immunocompromised patients [5].

Effects of immunosuppressive therapy

Corticosteroids represent one of the most potent modifiable risk factors for postoperative infection, including PJI, exerting broad immunosuppressive effects and impairing wound healing through reduced collagen synthesis, impaired angiogenesis, and decreased fibroblast proliferation. Observational studies consistently demonstrate that chronic corticosteroid use, particularly at doses exceeding 10 mg/day of prednisone equivalent, approximately doubles the risk of postoperative infection [6]. Biologic agents targeting specific inflammatory pathways carry significant infection risks: a Veterans Affairs case-control study demonstrated that perioperative continuation of biologic therapy increased the odds of PJI by 3.46-fold (95% CI 1.11-10.78) compared with patients who interrupted biologics perioperatively [7]. TNF-α inhibitors are essential for macrophage activation, neutrophil recruitment, and granuloma formation, and their blockade impairs these protective responses; case reports have documented difficult-to-eradicate PJIs in patients receiving anti-TNF therapy, with some infections requiring multiple revision surgeries [8]. Newer biologics used in psoriasis and psoriatic arthritis, IL-17 inhibitors (secukinumab, ixekizumab, and brodalumab), IL-23 inhibitors (guselkumab, risankizumab, and tildrakizumab), and the IL-12/23 inhibitor ustekinumab modulate axes central to psoriatic inflammation. Direct evidence regarding their perioperative PJI risk is limited; current guidance manages them as other biologics, with temporary interruption timed to the end of the dosing cycle [4]. The 2022 ACR/AHKS guideline recommends continuing methotrexate perioperatively for most patients, as the benefits of maintaining disease control outweigh infection risks, while temporarily interrupting biologic and JAK inhibitor therapies [4].

Clinical risk stratification

**Figure 1:**
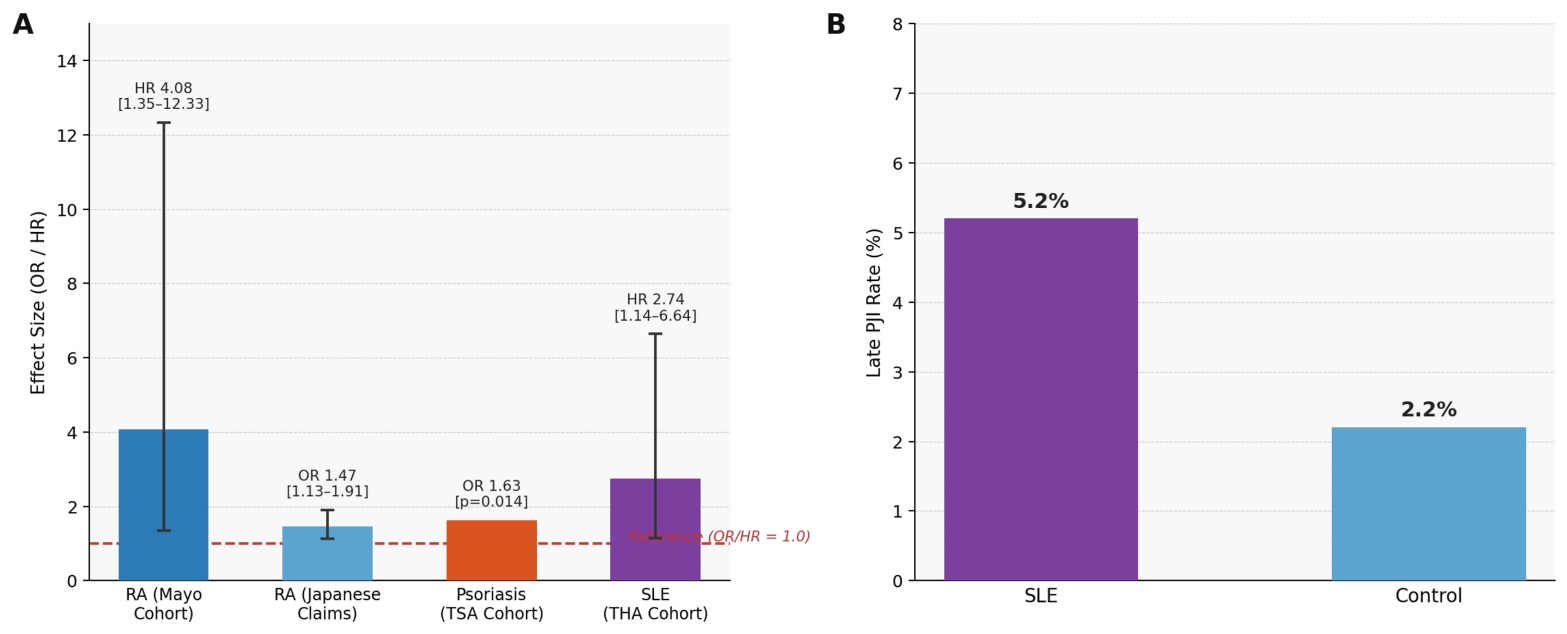
PJI risk in autoimmune and inflammatory diseases compared with non-autoimmune controls. (A) Effect sizes (ORs or HRs with 95% confidence intervals) for PJI risk across four cohorts: RA from the Mayo Clinic cohort (HR 4.08) [2]; RA from a Japanese nationwide claims database (OR 1.47) [9]; psoriasis undergoing total shoulder arthroplasty (OR 1.63) [3]; and SLE undergoing total hip arthroplasty (HR 2.74) [5]. (B) Absolute late PJI rates after total hip arthroplasty in SLE patients (5.2%) versus matched controls (2.2%) [5]. Estimates derive from separate analyses and are not pooled. PJI, periprosthetic joint infection; RA, rheumatoid arthritis; SLE, systemic lupus erythematosus; HR, hazard ratio; OR, odds ratio; CI, confidence interval. Image credit: Created by the authors using Python (Python Software Foundation, Wilmington, DE, USA) and the Matplotlib library (Matplotlib Development Team, USA).

Accurate risk stratification is essential for informed decision-making and implementation of preventive strategies. The Mayo Clinic cohort study by Bongartz et al. followed 462 RA patients undergoing 657 total hip or knee arthroplasties and documented a PJI incidence of 3.7% during a mean follow-up of 4.3 years, with a hazard ratio of 4.08 (95% CI 1.35-12.33) compared with matched osteoarthritis controls [2]. A Japanese nationwide medical claims database study of 9,048 matched RA-osteoarthritis pairs undergoing primary total knee arthroplasty found an odds ratio of 1.47 (95% CI 1.13-1.91) for in-hospital PJI [9]. Parel et al. analyzed 89,321 patients undergoing primary total shoulder arthroplasty and found that psoriasis patients demonstrated an odds ratio of 1.63 (p = 0.014) for 90-day PJI [3]. Among 325 SLE patients undergoing primary total hip arthroplasty matched to 325 controls, late PJI occurred in 5.2% of SLE patients compared with 2.2% of controls, yielding a hazard ratio of 2.74 (95% CI 1.14-6.64) [5]. The relative and absolute risks across conditions are compared in Figure 1.

**Figure 2:**
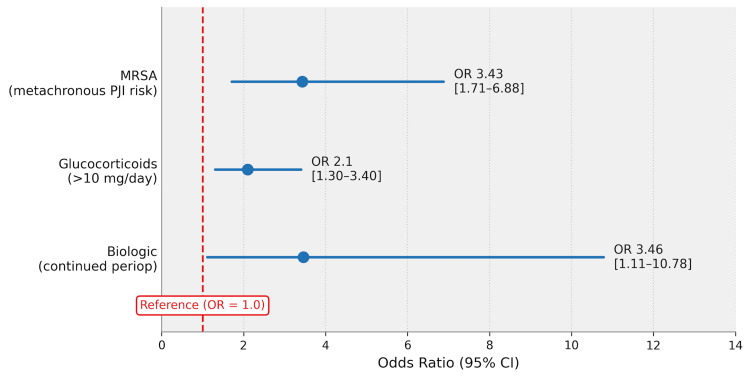
Forest plot of PJI risk factors in autoimmune and immunosuppressed patients. Perioperative continuation of biologic therapy demonstrates the highest odds ratio (OR 3.46, 95% CI 1.11–10.78) [7], followed by MRSA colonization (OR 3.43, 95% CI 1.71–6.88) [10], and chronic glucocorticoid use exceeding 10 mg/day (OR 2.1, 95% CI 1.30–3.40) [6]. Estimates derive from separate analyses and are not pooled. PJI, periprosthetic joint infection; MRSA, methicillin-resistant *Staphylococcus aureus*; OR, odds ratio; CI, confidence interval. Image credit: Created by the authors using Python (Python Software Foundation, Wilmington, DE, USA) and the Matplotlib library (Matplotlib Development Team, USA).

Immunosuppressive medication regimens represent critical modifiable risk factors. Perioperative continuation of biologic therapy increases PJI odds by 3.46-fold [7], and chronic corticosteroid use exceeding 10 mg/day approximately doubles infection risk [6]. Disease activity at surgery is another important consideration, as active inflammation is associated with elevated inflammatory markers, impaired wound healing, and potentially greater immunosuppressive requirements. *Staphylococcus aureus* colonization, particularly with methicillin-resistant *Staphylococcus aureus* (MRSA), is a potent risk factor: a meta-analysis by Li et al. demonstrated that MRSA colonization confers an odds ratio of 3.43 (95% CI 1.71-6.88) for metachronous PJI in patients with multiple prosthetic joints [10]. Given the increased *Staphylococcus aureus* colonization reported in subsets of psoriasis patients, preoperative screening and decolonization protocols warrant strong consideration. Key modifiable risk factors and their effect sizes are displayed in Figure 2.

These estimates should be interpreted with caution because the underlying studies are heterogeneous. They differ in design (prospective and retrospective cohorts, registry and claims analyses, and matched case-control studies); in population, joint, and procedure type; follow-up duration; PJI case definitions; and degree of confounder adjustment. In addition, hazard ratios and odds ratios measure different quantities, relative event rates over time versus the odds of an outcome, and should not be interpreted as directly comparable or combined into a pooled estimate. The values presented here, and those displayed in Figures 1-2, are drawn from separate analyses and are summarized descriptively rather than as a quantitative synthesis.

Clinical risk stratification should integrate disease-specific risk (RA > SLE > psoriasis based on available evidence), medication-related risk (biologics > corticosteroids > conventional DMARDs), disease activity, traditional risk factors, and colonization status. High-risk patients may benefit from enhanced preventive measures, including extended antibiotic prophylaxis, decolonization protocols, optimization of nutritional status and glycemic control, and consideration of staged procedures when feasible. Patient counseling should be thorough and individualized, emphasizing that while these conditions and their treatment increase infection risk, the absolute risk remains relatively low in most cases, and the functional benefits of arthroplasty typically outweigh infection risks.

Microbiology

Common Pathogens

*Staphylococcus aureus* and coagulase-negative staphylococci (primarily *Staphylococcus epidermidis*) account for more than 50% of PJI cases in general reviews [1], and these organisms remain the major pathogens in RA cohorts as well [11]. Staphylococcal virulence characteristics, adherence to prosthetic surfaces, biofilm formation, and production of enzymes and toxins encounter reduced host resistance in immunosuppressed patients, potentially facilitating more rapid biofilm establishment and clinical infection. MRSA PJI is associated with higher treatment-failure rates, an increased need for prosthesis removal, and worse functional outcomes compared with methicillin-susceptible *Staphylococcus aureus* infections, with colonization conferring an odds ratio of 3.43 (95% CI 1.71-6.88) for metachronous infection [10]. Streptococcal species account for approximately eight to 12% of PJI cases and are often associated with hematogenous seeding from dental, skin, soft-tissue, or respiratory sources. Gram-negative organisms, including* Escherichia coli* and *Pseudomonas aeruginosa*, account for approximately eight to 10% of cases and carry higher treatment-failure rates.

Culture-Negative PJI

Culture-negative infection appears to occur with increased frequency in RA-associated PJI. A single-center retrospective case series of 35 RA patients reported culture-negative infection in roughly 28%-38% of cases [12]; the same series reports this figure inconsistently (28.6% in its microbiological breakdown and discussion versus 38.4% in its abstract and conclusion), and the elevated rate is partly attributable to frequent pre-admission antibiotic exposure, which was present in nearly half of the patients. This range is broadly consistent with the 27%-37% described for PJI in systemic inflammatory disease. The elevated rate likely reflects prior antibiotic exposure, fastidious or slow-growing organisms missed by standard culture, biofilm-associated bacteria that resist culture, and altered immune responses affecting clinical presentation. A high culture-negative rate necessitates reliance on clinical criteria, synovial fluid analysis, histopathology, and molecular diagnostics, and often requires broad-spectrum empiric therapy without the ability to narrow the antibiotic spectrum based on culture results.

Opportunistic and Atypical Organisms

Unusual and opportunistic organisms may occasionally be encountered in this population. As a rare but illustrative example, a case report described successful treatment of *Mycobacterium ulcerans* prosthetic joint infection in an RA patient [13]; such single reports highlight the possibility of atypical pathogens but should not be interpreted as evidence of a general microbiologic pattern. The comparative organism distributions for general versus autoimmune PJI populations are summarized in Table 1.

**Table 1:** Comparative microbiology of PJI: general population vs. autoimmune/immunosuppressed patients *Culture-negative rate of ~28%–38% reported in a single-center case series of 35 RA patients [12]; the source reports the rate inconsistently (28.6% vs 38.4%), and the elevated rate is partly attributable to pre-admission antibiotic use. MSSA, methicillin-susceptible *Staphylococcus aureus*; MRSA, methicillin-resistant *Staphylococcus aureus*; RA, rheumatoid arthritis; PJI, periprosthetic joint infection. Table credit: Original table created by the authors.

Organism	General PJI (% of isolates)	RA/Autoimmune PJI (% of isolates)	Clinical implications
*Staphylococcus aureus* (MSSA)	28	30	Most common pathogen: MRSA screening in high-risk patients [1,10,11]
Coagulase-negative staphylococci	25	28	Often biofilm-forming; may require prosthesis removal [1,11]
MRSA	8	10	OR 3.43 (1.71–6.88) for metachronous PJI; higher treatment failure [10]
*Streptococcus *spp.	12	8	Often hematogenous; evaluate for distant infection sources [1]
Gram-negative rods	10	8	Associated with wound complications; higher treatment failure [1,11]
Culture-negative	10	~28%–38%*	Elevated in an RA case series [12]; consider novel synovial biomarkers (alpha-defensin, leukocyte esterase) and molecular diagnostics [14]

Perioperative management

Optimal perioperative management requires multidisciplinary coordination between orthopaedic surgery, rheumatology, and infectious disease to balance infection-risk reduction with maintenance of disease control. The 2022 ACR/AHKS guideline provides the most current evidence-based framework for perioperative antirheumatic medication management, though most recommendations remain conditional due to limited high-quality randomized controlled trial evidence [4]. Perioperative recommendations for each major drug class are summarized in Table 2.

**Table 2:** Perioperative management of immunosuppressive medications in patients undergoing total joint arthroplasty TNF, tumor necrosis factor; IL, interleukin; JAK, Janus kinase; OR, odds ratio; PJI, periprosthetic joint infection; ACR, American College of Rheumatology; AHKS, American Association of Hip and Knee Surgeons. Table credit: Original table created by the authors based on data from the 2022 ACR/AHKS guideline [4] and cited references.

Medication class	Specific agents	Perioperative recommendation	Evidence level	Key evidence
Corticosteroids	Prednisone, methylprednisolone	Continue; stress-dose for ≥20 mg/day	Moderate	Chronic >10 mg/day doubles infection risk [6]
TNF-α inhibitors	Infliximab, etanercept, adalimumab, certolizumab, golimumab	Temporarily interrupt; surgery at end of dosing cycle; resume after wound healing (~14 days)	Moderate	Perioperative continuation OR 3.46 [1.11–10.78] for PJI [4,7]
IL-6 inhibitors	Tocilizumab, sarilumab	Interrupt; end of dosing cycle; resume after wound healing	Low-Moderate	Extrapolated from TNF-inhibitor data [4]
IL-17 inhibitors	Secukinumab, ixekizumab, brodalumab	Interrupt; end of dosing cycle; resume after wound healing	Low	Limited direct evidence [4]
IL-23/IL-12/23 inhibitors	Guselkumab, risankizumab, tildrakizumab, ustekinumab	Interrupt; end of dosing cycle; resume after wound healing	Low	Limited direct PJI evidence; managed as other biologics per guideline [4]
B-cell depleting agents	Rituximab	Interrupt; surgery when B-cell counts recover (typically 6–12 months post-dose)	Low-Moderate	Profound immunosuppression; extended duration of effect [4]
Methotrexate	Methotrexate	Continue perioperatively	Moderate	Benefits of disease control outweigh infection risk [4]
Leflunomide	Leflunomide	Interrupt one to two days before surgery; resume after wound healing	Low	Long half-life; individualized [4]
Sulfasalazine/Hydroxychloroquine	Sulfasalazine, hydroxychloroquine	Continue perioperatively	Low-Moderate	Minimal immunosuppression; low infection risk [4,15]
JAK inhibitors	Tofacitinib, baricitinib, upadacitinib	Interrupt; end of dosing cycle; resume after wound healing	Low-Moderate	Emerging evidence [4,16]

Preoperative optimization

Preoperative optimization should occur well in advance of planned surgery. Disease activity should be in remission or low disease activity at surgery to minimize both infection risk and postoperative flare risk. Nutritional status should be assessed via serum albumin, prealbumin, and total lymphocyte count, with supplementation for identified deficiencies, as malnutrition impairs wound healing and immune function. Glycemic control should target HbA1c below 7% to 8% before elective surgery. Smoking cessation should be strongly encouraged, with a goal of at least four to six weeks of abstinence. MRSA screening with nasal swab culture should be considered in high-risk patients, particularly those with psoriasis or other chronic skin conditions associated with increased *Staphylococcus aureus* colonization; decolonization with intranasal mupirocin and chlorhexidine body washes for five to seven days before surgery represents a potentially high-yield intervention given the 3.43-fold increased odds of metachronous PJI associated with MRSA colonization [10]. Dental evaluation and treatment of active infections should be completed before elective arthroplasty, as dental sources represent a potential nidus for hematogenous PJI [17].

Medication management

Biologic agents should generally be interrupted perioperatively per current evidence [7] and guideline recommendations [4]; the optimal approach depends on drug half-life, individual disease activity, and flare risk, and current guidance is conditional. Surgery should be scheduled at the end of the dosing cycle to allow maximum drug clearance, and resumption should be delayed until adequate wound healing is confirmed, typically 10 to 14 days postoperatively in uncomplicated cases. The same end-of-cycle timing approach is applied to the newer psoriasis biologics (IL-17, IL-23, and IL-12/23 inhibitors) and to JAK inhibitors, though direct PJI evidence for these agents is limited [4]. Methotrexate should be continued perioperatively per the 2022 ACR/AHKS guideline [4], a change from the 2017 recommendation to withhold, based on evidence that continuation does not significantly increase infection risk and that discontinuation may precipitate flares. Corticosteroids should generally be continued at the patient's current dose to avoid adrenal insufficiency and disease flare, with stress-dose supplementation considered for doses ≥20 mg/day; the goal should be minimizing chronic corticosteroid doses preoperatively when feasible, recognizing that doses exceeding 10 mg/day approximately double infection risk [6].

Intraoperative strategies

Intraoperative antibiotic prophylaxis should follow standard guidelines for prosthetic joint surgery, typically cefazolin two grams (three grams for patients >120 kg) administered within 60 minutes before incision, with redosing every four hours during prolonged procedures. For patients with MRSA colonization or high MRSA risk, vancomycin should be added or substituted. Surgical technique should emphasize meticulous hemostasis, gentle tissue handling, and anatomic wound closure.

Postoperative surveillance

Postoperative wound surveillance should be intensive in immunosuppressed patients, with a low threshold for wound exploration and culture, as early detection and treatment of superficial infection may prevent progression to deep PJI. Multidisciplinary coordination throughout the perioperative period, including preoperative multidisciplinary conferences for high-risk patients, can facilitate coordinated care planning and may improve outcomes [17].

Future directions

Substantial knowledge gaps remain. Mechanistic investigations are needed to define the pathways linking skin-barrier dysfunction, altered microbiome, and immune dysregulation to PJI risk, using techniques including single-cell RNA sequencing and microbiome profiling. Prospective cohort studies with standardized PJI definitions and long-term follow-up are needed to refine risk estimates, as most existing evidence is retrospective. Of particular relevance to dermatology, future studies should investigate psoriasis-specific variables, PASI score, body-surface-area involvement, scalp involvement, and nail disease, and their relationship with PJI, since current evidence does not stratify risk by psoriasis severity or phenotype. Microbiome studies characterizing the lesional and peri-incisional cutaneous microbiome prior to arthroplasty may identify modifiable predictors of infection. Randomized controlled trials of perioperative medication management are urgently needed, as most ACR/AHKS recommendations are conditional [4]. Molecular diagnostic techniques, including next-generation sequencing and multiplex PCR, hold promise for improving the diagnosis of culture-negative PJI, and novel biomarkers, including synovial fluid alpha-defensin and leukocyte esterase, require validation in these populations [14].

## Conclusions

Autoimmune and inflammatory diseases increased PJI risk through convergent mechanisms whose relative contributions differed by disease: skin-barrier dysfunction (principally in psoriasis), systemic immune dysregulation, and immunosuppressive-therapy effects. Quantitative risk estimates revealed hazard ratios of 1.47 to 4.08 for RA, 1.63 for psoriasis, and 2.74 for SLE compared with non-autoimmune controls, with perioperative continuation of biologic therapy conferring an additional 3.46-fold increase in odds; these estimates were derived from heterogeneous studies and were not directly comparable. The microbiologic profile was distinct, with culture-negative infection common in RA-associated PJI (roughly 28%-38% in a single-center case series); elevated MRSA prevalence carried a 3.43-fold increased odds of metachronous infection; and the potential for atypical and opportunistic organisms, all of which necessitated modified diagnostic and therapeutic approaches. Most available evidence was retrospective and observational, which limited causal inference.

Evidence-based perioperative management requires multidisciplinary coordination to balance infection-risk reduction with maintenance of disease control. Current guidance supports temporarily interrupting biologic agents with surgery scheduled at the end of the dosing cycle, continuing methotrexate in most patients, and applying individualized management to other immunosuppressive medications. Preoperative optimization encompassing disease-activity control, nutritional status, glycemic control, smoking cessation, and MRSA screening forms the foundation for infection prevention, complemented by meticulous surgical technique, appropriate antibiotic prophylaxis, and intensive postoperative wound surveillance. Most current guideline recommendations remain conditional due to limited high-quality evidence, underscoring the need for prospective cohort studies, randomized controlled trials, and mechanistic investigations to fill existing knowledge gaps.
